# Evaluating opioid and analgesic regimens for post-operative pain management in gynecological surgery: A comparative study

**DOI:** 10.6026/973206300223148

**Published:** 2026-05-31

**Authors:** Ashis Tiwari, Shambhavi Soni, Pradeep Rathore

**Affiliations:** 1 Department of General Medicine, Government Medical College, Datia, Madhya Pradesh, India; 2 Department of Obstetrics and Gynaecology, Government Medical College, Datia, Madhya Pradesh, India; 3 Department of General Surgery, Government Medical College, Datia, Madhya Pradesh, India

**Keywords:** Post-operative pain, gynecological surgery, opioid analgesics, multimodal analgesia, pain management, visual analog scale (VAS)

## Abstract

Effective post-operative pain control remains a crucial component of recovery following gynecological surgery, with opioid-related adverse effects posing significant clinical challenges. Multimodal analgesia, integrating multiple drug classes acting through different mechanisms, offers a promising approach to enhance pain relief while reducing opioid dependency. Hence, this prospective comparative study included 150 patients undergoing elective gynecological surgeries divided into two groups: Group A received opioid-based analgesic regimens and Group B was treated using multimodal analgesia combining opioids and non-opioid agents. Pain intensity was assessed at scheduled post-operative intervals using the Visual Analog Scale (VAS), alongside documentation of adverse effects. Thus, we show that multimodal analgesic regimens provide safer and more effective post-operative analgesia than traditional opioid-based approaches for gynecological surgical patients.

## Background:

Patients having gynecological surgeries still worry a lot about post-operative discomfort. In addition to ensuring the patient's comfort, prompt movement, less surgical complications and a shortened length of hospital stay are also outcomes of well-managed pain. Complications such as persistent post-operative pain syndromes, longer hospital stays, higher healthcare expenditures and slowed recovery times might result from inadequate pain management after surgery [1]. Clinical practice still faces the difficulty of providing appropriate post-operative analgesia, despite improvements in surgical methods and anesthetic administration. Failure to use suitable analgesic techniques results in moderate to severe pain experienced by more than 60-80% of patients after surgical operations, according to studies [2]. Because of their powerful analgesic effects, opioids have long been regarded as the gold standard for managing post-operative pain. It is usual practice to provide opioids like morphine, fentanyl and tramadol to patients immediately after surgery in order to alleviate their pain [3]. Opioids mainly modify pain perception and suppress nociceptive transmission via acting on μ-opioid receptors in the central nervous system. Although opioid-based analgesic regimens are effective, they are also linked to a number of side effects, such as vomiting, nausea, constipation, respiratory depression and vomiting, pruritus, urinary retention and sedation. These side effects may delay recovery, prolong hospital stay and negatively affect patient satisfaction. Additionally, increasing concerns regarding opioid misuse, tolerance and dependence have encouraged clinicians to explore alternative strategies for post-operative pain management [4]. When it comes to managing pain during surgery, the idea of multimodal analgesia has recently become quite popular. The goal of multimodal analgesia is to alleviate pain by using a variety of analgesic drugs and procedures that each targets a distinct route in the brain.

Acetaminophen, local anesthetics, regional anesthesia methods, low-dose opioids, nonsteroidal anti-inflammatory medicines (NSAIDs) and other medications may be part of this strategy. By targeting multiple pathways involved in pain transmission, multimodal analgesia aims to enhance analgesic efficacy while minimizing the dose and adverse effects of individual drugs, particularly opioids [5]. Multiple clinical trials have shown that compared to opioid-only regimens, multimodal analgesic regimens considerably decrease the severity of post-operative pain and opioid use. Peng *et al.* highlighted that multimodal analgesia improves post-operative recovery by reducing opioid-related side effects and facilitating early ambulation and functional recovery [6]. Similarly, Moulder *et al.* reported that multimodal pain management protocols in surgical patients were associated with improved analgesic outcomes and reduced opioid requirements [7]. In gynecological surgeries, which include procedures such as hysterectomy, oophorectomy and myomectomy, it is crucial to effectively manage post-operative pain since insufficient pain relief might hinder breathing, postpone mobility and raise the likelihood of thromboembolic complications [8]. Gynecological surgical procedures often involve significant tissue manipulation and visceral pain, making post-operative analgesia challenging. Traditionally, opioids have been widely used in this patient population; however, opioid-related adverse effects such as post-operative nausea and vomiting (PONV) are especially common among female patients [9]. Multimodal analgesia provides superior postoperative pain control with reduced opioid consumption and fewer adverse effects compared to opioid-based regimens, leading to higher patient satisfaction in gynecological surgeries [10]. Therefore, it is of interest to evaluate the efficacy of opioid-based and multimodal analgesic regimens in reducing post-operative pain in women having gynecological surgeries.

## Materials and Methodology:

## Study design and setting:

This prospective comparison research was carried out in the Obstetrics and Gynecology Department in conjunction with the Anesthesiology Department at a tertiary care teaching hospital. Once the Institutional Ethics Committee gave its approval, the research could begin and continued for a full year. Before anybody could be a part of the research, they had to provide their written informed permission.

## Study population and sample size:

A total of 150 female patients scheduled to undergo elective gynecological surgeries under spinal or general anesthesia were included in the study.

The participants were randomly allocated into two equal groups consisting of 75 patients each:

[1] Group A (n = 75): Patients receiving opioid-based analgesic regimen for post-operative pain management.

[2] Group B (n = 75): Patients receiving multimodal analgesic regimen consisting of a combination of non-opioid analgesics with limited opioid use when required.

## Inclusion criteria:

[1] Female patients aged 18-60 years.

[2] Patients undergoing elective gynecological surgeries such as abdominal hysterectomy, vaginal hysterectomy, ovarian cystectomy, myomectomy, or other similar procedures.

[3] Patients classified as American Society of Anesthesiologists (ASA) physical status I or II.

[4] Patients who provided informed consent for participation in the study.

## Exclusion criteria:

[1] Patients with known hypersensitivity or contraindications to opioids, NSAIDs, or paracetamol.

[2] Patients with chronic pain disorders or those receiving long-term opioid therapy.

[3] Patients with severe hepatic, renal, or cardiovascular disease.

[4] Patients with psychiatric illness or cognitive impairment interfering with pain assessment.

[5] Emergency surgeries or patients requiring post-operative intensive care.

## Analgesic protocol:

## Group A (Opioid-Based Analgesia):

Patients in this group received intravenous opioids for post-operative pain control. The standard regimen consisted of intravenous tramadol (50-100 mg) administered every 6-8 hours as required for pain relief. Rescue analgesia with intravenous morphine (2-3 mg) was administered when the pain score exceeded the predefined threshold.

## Group B (Multimodal analgesia):

Patients in this group received a combination of analgesic medications acting through different mechanisms, which included:

[1] Intravenous paracetamol (1 g) every 6 hours

[2] Nonsteroidal anti-inflammatory drugs (NSAIDs) such as diclofenac (75 mg) every 12 hours 

[3] Low-dose opioid (tramadol 50 mg) administered only if required as rescue analgesia

This multimodal regimen aimed to provide adequate analgesia while minimizing opioid consumption.

## Data collection:

Age, BMI, operation type and total time under the knife were among the demographic data reported. Using a Visual Analog Scale (VAS) from 0 (no pain) to 10 (the greatest possible agony), post-operative pain was evaluated.

Pain assessment was performed at the following post-operative intervals:

[1] 2 hours

[2] 6 hours

[3] 12 hours

[4] 24 hours

## In addition to pain scores, the following parameters were recorded:

[1] Requirement of rescue analgesia

[2] Incidence of post-operative nausea and vomiting (PONV)

[3] Sedation level

[4] Additional side effects of opioids include itching, decreased respiratory function and constipation.

[5] Time to first mobilization

## Outcome measures:

[1] Comparison of post-operative pain intensity between the two groups using VAS scores.

[2] Requirement for additional or rescue analgesia.

[3] Incidence of opioid-related adverse effects.

[4] Patient comfort and early mobilization following surgery.

## Statistical analysis:

Microsoft Excel was used to input all of the obtained data and SPSS version 26.0 was used for analysis. Categorical data were represented as frequency and percentage, whilst continuous variables were shown as mean ± standard deviation. In order to compare the two groups, Independent t-test for continuous variables and Chi-square test or Fisher’s exact test for categorical variables was used. A p-value < 0.05 was considered statistically significant.

## Results:

The research comprised 150 individuals who were set to have gynecological surgery. They were split into two equal groups: Group A (Opioid-Based Analgesic Regimen, n = 75) and Group B (Multimodal Analgesic Regimen, n = 75). The results were analyzed with respect to demographic characteristics, post-operative pain scores and incidence of post-operative adverse effects. Patients in both groups were categorized according to age and the kind of gynecological surgery performed (Table 1). The age range of 31-45 years comprised the bulk of patients in both the opioid group (42.7%) and the multimodal group (40.0%). The surgical treatment that was done most often in both groups was abdominal hysterectomy. Participants' baseline characteristics were similar across the two groups, as there were no statistically significant variations in the distribution of ages or types of surgeries (p > 0.05). Table 2 compares post-operative pain intensity between the two groups using the Visual Analog Scale (VAS) at different post-operative time intervals. At 2 hours post-operatively, a higher proportion of patients in the multimodal analgesia group experienced mild pain (42.7%) compared to the opioid group (24.0%), while severe pain was more common in the opioid group (22.7% vs 10.6%).

Similar trends were observed at 6 hours and 24 hours, where the multimodal group consistently reported lower pain severity. These differences were statistically significant (p < 0.05), indicating that multimodal analgesia provided more effective post-operative pain control than opioid-based analgesia alone. The two groups' post-operative adverse effects are compared in Table 3. The most prevalent side effects in both groups were nausea and vomiting, although the opioid-based analgesia group had a much higher incidence of 37.3% compared to the multimodal group's 20.0% (p = 0.02). Similarly, sedation and constipation were more frequently reported in the opioid group. There was a statistically significant difference between the two groups, with 56.0% of patients in the multimodal analgesia group reporting no side effects and 29.3% in the opioid group (p = 0.001). Based on these results, it seems that multimodal analgesia has less opioid-related side effects and better tolerance.

## Discussion:

The treatment of post-operative pain is an important issue for patients having gynecological surgeries. Post-operative discomfort that is not well managed might cause a delay in mobility, prolonged hospitalization, increased post-operative complications and reduced patient satisfaction. Traditionally, opioid-based analgesic regimens have been widely used because of their potent analgesic effects; however, alternative techniques, such multimodal analgesia, have been investigated because to the harmful consequences, which include nausea, vomiting, drowsiness, respiratory depression and the danger of dependence. In order to obtain better pain management with less opioid use, multimodal analgesia makes use of many analgesic drugs that work via diverse pathways. Post-operative pain management was better with the multimodal analgesic regimen than with the opioid-based regimen in this trial of 150 patients having gynecological surgery. At various post-operative intervals, patients who received multimodal analgesia reported substantially reduced post-operative pain levels and needed fewer rescue medications. These results add to the growing body of research suggesting that a multimodal approach to pain management after surgery may improve outcomes while decreasing the need for opioids [11]. In patients having elective gynecological surgeries, Ahmed and Amjad (2025) contrasted traditional opioid-based analgesia with multimodal analgesia. At various stages after surgery, the patients in the group that received multimodal analgesia reported much less pain on the Visual Analog Scale (VAS). The multimodal group also had a decreased incidence of nausea and vomiting and a much lower opioid use in the first 24 hours compared to the opioid-only group (6.4 mg versus 13 mg). Consistent with the current study's findings, these studies show that multimodal analgesic treatments are helpful for gynecological surgeries [1]. Similarly, patients who received multimodal analgesia after laparoscopic gynecological surgery reported less pain, used fewer opioids and had a faster recovery time, according to a randomized controlled experiment, earlier ambulation and faster bowel recovery compared with patients receiving conventional opioid-based analgesia. The study also reported higher patient satisfaction in the multimodal analgesia group, further supporting the benefits of opioid-sparing analgesic strategies [12]. Another study examining multimodal analgesic protocols in gynecologic laparotomy patients demonstrated that the introduction of a multimodal pain management strategy significantly reduced post-operative pain scores and shortened hospital stay. Patients treated with multimodal analgesia experienced improved recovery parameters without increasing post-operative complications [13]. The present study also found a higher incidence of opioid-related adverse effects such as nausea, vomiting, sedation and constipation in the opioid-based analgesia group compared with the multimodal group. These findings are in agreement with previous clinical studies which have demonstrated that opioid use is commonly associated with gastrointestinal and central nervous system side effects. Multimodal analgesia reduces these complications by decreasing the amount of opioids required for adequate pain control. A randomized controlled clinical trial conducted by Lopez *et al.*. (2019) evaluating multimodal analgesic techniques in gynecological laparoscopy reported significantly improved post-operative pain control when a multimodal approach was used. The combination of different analgesic modalities provided better analgesia compared with single-technique approaches and improved post-operative recovery [14]. Similarly, studies evaluating opioid-sparing multimodal regimens in gynecologic pelvic reconstructive surgery have demonstrated improved post-operative pain outcomes and reduced opioid consumption when non-opioid analgesics such as acetaminophen and NSAIDs were incorporated into post-operative pain protocols [15, 16]. The capacity to target several components of the pain pathway is the mechanism by which multimodal analgesia improves efficacy. Regional anesthetic procedures obstruct nerve conduction at the surgical site, non-opioid analgesics like NSAIDs decrease peripheral inflammation and opioids mainly operate on CNS receptors to limit nociceptive transmission. By combining these different mechanisms, multimodal analgesia produces a synergistic effect that enhances pain relief while minimizing the adverse effects associated with high-dose opioids. Furthermore, the use of multimodal analgesic techniques is highly supported by the Enhanced Recovery After Surgery (ERAS) concept.

The goal of the ERAS protocols is to enhance surgical outcomes by reducing opioid usage, increasing perioperative analgesia and encouraging early mobility. Multiple studies have shown that gynecological surgery patients benefit from multimodal analgesia within ERAS pathways, which leads to faster recovery, shorter hospital stays and higher patient satisfaction. Despite the advantages of multimodal analgesia, certain limitations must be considered. Variability in drug combinations, differences in surgical procedures and variations in patient characteristics may influence analgesic outcomes. Additionally, The results may not be applicable to a broader population since this research only included 150 patients and was carried out at one facility. Multimodal analgesia has to be further validated in larger multicenter randomized controlled studies in order to treat varied surgical groups effectively. In sum, the results corroborate those of previous research and provide credence to the increasing trend of using a combination of analgesic modalities to alleviate post-operative pain. Improving surgical pain management and minimizing opioid-related complications may be achieved by the integration of multimodal analgesia into standard clinical practice.

## Conclusion:

Data shows that multimodal analgesic regimens work better than opioid-based regimens when managing pain after gynecological surgery. There was a considerable decrease in pain ratings, opioid needs and opioid-related side effects in patients who received multimodal analgesia. Data recommends the introduction of multimodal analgesia as a regular method in pain management regimens in order to improve patient outcomes and post-operative recovery after gynecological procedures.

## Figures and Tables

**Table 1:** Demographic characteristics of study participants

**Variable**	**Opioid-Based Regimen (n=75)**	**Multimodal Regimen (n=75)**	**p-value**
Age Group (years)			0.68
18-30	18 (24.0%)	20 (26.7%)	
31-45	32 (42.7%)	30 (40.0%)	
46-60	25 (33.3%)	25 (33.3%)	
Type of Surgery			0.74
Abdominal hystere ctomy	28 (37.3%)	30 (40.0%)	
Vaginal hysterectomy	20 (26.7%)	18 (24.0%)	
Ovarian cystectomy	15 (20.0%)	14 (18.7%)	
Myomectomy	12 (16.0%)	13 (17.3%)	

**Table 2:** Comparison of post-operative pain scores (VAS)

**Time after Surgery**	**Opioid-Based Regimen (n=75)**	**Multimodal Regimen (n=75)**	**p-value**
**2 hours**			
Mild (VAS 1-3)	18 (24.0%)	32 (42.7%)	
Moderate (VAS 4-6)	40 (53.3%)	35 (46.7%)	
Severe (VAS 7-10)	17 (22.7%)	8 (10.6%)	0.02
**6 hours**			
Mild	22 (29.3%)	40 (53.3%)	
Moderate	38 (50.7%)	30 (40.0%)	
Severe	15 (20.0%)	5 (6.7%)	0.01
**24 hours**			
Mild	30 (40.0%)	52 (69.3%)	
Moderate	35 (46.7%)	20 (26.7%)	
Severe	10 (13.3%)	3 (4.0%)	0.004

**Table 3:** Incidence of post-operative adverse effects

**Adverse Effect**	**Opioid-Based Regimen (n=75)**	**Multimodal Regimen (n=75)**	**p-value**
Nausea and vomiting	28 (37.3%)	15 (20.0%)	0.02
Sedation	20 (26.7%)	9 (12.0%)	0.03
Constipation	16 (21.3%)	8 (10.7%)	0.04
Pruritus	9 (12.0%)	4 (5.3%)	0.14
No adverse effects	22 (29.3%)	42 (56.0%)	0.001
